# Proteomics and Toxicity Analysis of Spinal-Cord Primary Cultures upon Hydrogen Sulfide Treatment

**DOI:** 10.3390/antiox7070087

**Published:** 2018-07-10

**Authors:** Viviana Greco, Alida Spalloni, Victor Corasolla Carregari, Luisa Pieroni, Silvia Persichilli, Nicola B. Mercuri, Andrea Urbani, Patrizia Longone

**Affiliations:** 1 Institute of Biochemistry and Clinical Biochemistry, Università Cattolica del Sacro Cuore, 00168 Rome, Italy; vivianagreco82@yahoo.it (V.G.); corasolla@gmail.com (V.C.C.); silvia.persichilli@unicatt.it (S.P.); andrea.urbani@unicatt.it (A.U.); 2 Department of Laboratory Diagnostic and Infectious Diseases, Fondazione Policlinico Universitario Agostino Gemelli-IRCCS, 00168 Rome, Italy; 3 Department of Experimental Neuroscience, Molecular Neurobiology Unit, Fondazione Santa Lucia, 00143 Rome, Italy; a.spalloni@hsantalucia.it; 4 Department of Experimental Neuroscience, Proteomics and Metabonomics Unit, Fondazione Santa Lucia-IRCCS, 00143 Rome, Italy; l.pieroni@hsantalucia.it; 5 Department of Systems Medicine, Policlinico Universitario “Tor Vergata”, University of Rome “Tor Vergata”, 00133 Rome, Italy; mercurin@med.uniroma2.it; 6 Department of Experimental Neuroscience, Experimental Neurology Unit, 00143 Rome, Italy

**Keywords:** apoptosis, hydrogen sulfide, motor neuron, necroptosis, proteomics

## Abstract

Hydrogen sulfide (H_2_S) is an endogenous gasotransmitter recognized as an essential body product with a dual, biphasic action. It can function as an antioxidant and a cytoprotective, but also as a poison with a high probability of causing brain damage when present at noxious levels. In a previous study, we measured toxic liquoral levels of H_2_S in sporadic amyotrophic lateral sclerosis (ALS) patients and in the familial ALS (fALS) mouse model, SOD1G93A. In addition, we experimentally demonstrated that H_2_S is extremely and selectively toxic to motor neurons, and that it is released by glial cells and increases Ca^2+^ concentration in motor neurons due to a lack of ATP. The presented study further examines the effect of toxic concentrations of H_2_S on embryonic mouse spinal-cord cultures. We performed a proteomic analysis that revealed a significant H_2_S-mediated activation of pathways related to oxidative stress and cell death, particularly the Nrf-2-mediated oxidative stress response and peroxiredoxins. Furthermore, we report that Na_2_S (a stable precursor of H_2_S) toxicity is, at least in part, reverted by the Bax inhibitor V5 and by necrostatin, a potent necroptosis inhibitor.

## 1. Introduction

In 2015 [1], we showed that hydrogen sulfide (H_2_S) reaches toxic levels in the cerebrospinal fluid (CSF) of sporadic amyotrophic lateral sclerosis (ALS) patients. Furthermore, we documented a correlation between the concentration of H_2_S and the site of disease onset, with significantly higher levels of H_2_S in the limb-onset group compared to the bulbar-onset group. In the same work, we demonstrated that (i) neuronal tissues from the familial ALS (fALS) mouse model, SOD1G93A contain high levels of H_2_S compared to the related controls; (ii) glial cells are the major source of H_2_S, since halting their growth in vitro resulted in a significant decrease in H_2_S concentration in the cell media; (iii) H_2_S is toxic to motor neurons, as compared to gamma-aminobutyric acid+ (GABA+) neurons, partly through the a-amino-3-hydroxy-5-methyl-4-isoxazole propionic acid (AMPA) receptor; and (iv) H_2_S increases Ca^2+^ concentration in motor neurons, triggered by a decrease in ATP production [1].

Hydrogen sulfide, like carbon monoxide and nitric oxide, is now recognized as a cellular gaseous mediator and a neurotransmitter [2,3,4]. It is produced via cysteine catabolism by the cytoplasmic enzymes, cystathionine-β-synthase (CBS) and cystathionine-γ-lyase (CSE), and also via 3-mercaptopyruvate catabolism by 3-mercaptopyruvate sulfurtransferase (3-MST) [5]. It is a challenging molecule. At physiological concentrations (nM to low-μM), H_2_S produces beneficial effects; however, when it reaches higher concentrations, as the one that we measured in the ALS patients, it can have harmful actions [1]. Indeed, on one hand, H_2_S showed protective abilities in cellular and animal models of Parkinson’s disease (PD) and Alzheimer’s disease (AD). It can revert 6-hydroxydopamine (6-OHDA)-induced neuronal loss, suggesting a protective effect of H_2_S in PD [6]. In AD, H_2_S showed the ability to ameliorate Aβ-induced cell apoptosis [7], improve spatial memory, and reduce the production of Aβ in the human amyloid precursor protein (Mo/HuAPP695swe) and a mutant human presenilin 1 (PS1-dE9) (APP/PS1) AD mouse model [8]. On the other hand, it is one of the major toxic gases with fatal effects if inhaled [9,10]. At high concentrations (300 μM and 1 mM), it causes an N-methyl-D-aspartate (NMDA) receptor-independent neuronal death via the activation of apoptotic mechanisms and the MEK/ERK pathway [11], as well as through a glutamate-mediated activation of calpains and the destabilization of the lysosomal membrane in cortical neurons [12].

As a follow-up to our previous work [1], here we examined the potential death mechanisms activated by H_2_S on mixed primary mouse spinal-cord cultures. In this work, we used the proteomic approach to examine (unravel) how protein expression changes in H_2_S-treated and H_2_S-untreated primary spinal-cord cultures down to the cellular level. The proteomic analysis highlighted a significant perturbation of the Nrf-2-mediated oxidative stress response, peroxiredoxins, and hypoxia-inducible factors. Moreover, we showed that, at relatively high concentrations (100 μM and 200 μM), H_2_S activated, in primary spinal-cord cultures, cellular pathways linked to apoptosis and necrosis. Specifically H_2_S-induced neuronal death was, in part, neutralized by the Bax inhibitor V5 and by necrostatin, a necroptosis inhibitor.

## 2. Material and Methods

### 2.1. Primary Spinal-Cord Culture

Mixed spinal cords were prepared from 13.5-day-old embryos of C57BL6xSJL mice housed at the Fondazione Santa Lucia animal facility (Protocol Number 93O/2017-PR; Spalloni et al., 2004). After dissection, the spinal cord cultures were plated on glass cover slips, and were maintained in Neurobasal medium supplemented with B-27 and 0.5 mM glutamine. The medium was replaced with Neurobasal supplemented with B-27, and was changed every 3 days [13].

### 2.2. Proteomic Analysis

Following the toxicity protocol, spinal-cord cultures were washed three times in PBS, and cell lysates were prepared by solubilizing the cells in RIPA buffer (150 mM NaCl, 1 mM EDTA, 0.5% sodium deoxycolate, 0.1% sodium dodecyl sulfate (SDS) SIGMA-Aldrich, Milan, Italy) with freshly added protease and phosphatase inhibitors (protease inhibitor cocktail; SIGMA).

Briefly, extracts derived from the spinal-cord culture were precipitated with a cold mix of ethanol, methanol, and acetone (ratio 2:1:1, *v*/*v*), then dissolved in 6 M urea and 100 mM Tris at pH 7.5, and digested 50:1 (*w*/*w*) with sequence-grade trypsin (Promega, Madison, WI, USA) at 37 °C overnight after reduction with 10 mM dithiothreitol (DTT) and alkylation with 20 mM Iodoacetamide (IAA). The reaction was stopped by adding Trifluoroacetic acid (TFA) to a final concentration of 0.1%.

Label-free proteomic analysis was performed, as previously described by Piras et al. [14,15], with some modifications. Firstly, 100 fmol/μL of digestion of enolase from *Saccharomyces cerevisiae* was added to each sample as an internal standard, and then, separation of tryptic peptides was performed on an ACQUITY MClass System (Waters Corporation, Waters S.p.A., Sesto San Giovanni, Italy). Each digested sample (0.25 µg) was loaded onto a Symmetry C18 5 μm, 180 μm × 20 mm precolumn (Waters Corporation, Waters S.p.A., Sesto San Giovanni, Italy), and was subsequently separated by a 90-min reversed-phase gradient at 300 nL/min (linear gradient, 2–85% CH_3_CN over 90 min) using an HSS T3 C18 1.8 μm, 75 μm × 150 mm nanoscale liquid chromatography (LC) column (Waters Corporation) maintained at 40 °C. The separated peptides were analyzed using a high-definition Synapt G2-Si mass spectrometer (Waters Corporation, Waters S.p.A., Sesto San Giovanni, Italy), directly coupled to the chromatographic system. Differential protein expression was evaluated with a data-independent acquisition (DIA) of shotgun proteomic analysis using expression configuration mode (MS^E^). The mass spectrometer operated in “expression mode” switching between low (4 eV) and high (15–40 eV) collision energies on the gas cell, using a scan time of 1.5 s per function over 50–2000 *m*/*z*. The processing of low and elevated energy, added to the data of the reference lock mass ([Glu1]-Fibrinopeptide B Standard, Waters Corporation, Waters S.p.A., Sesto San Giovanni, Italy), provided a time-aligned inventory of accurate mass-retention time components for both the low- and elevated-energy exact mass retention time (EMRT). Each sample was run in four technical replicates. 

### 2.3. Bioinfomatic Analysis

The analysis of differentially expressed proteins was performed according to Silva et al. [16] and Visser et al. [17]. Continuum LC-MS data from the four replicate experiments for each sample were processed for qualitative and quantitative analysis using the ProteinLynx Global Server version 3.0.1 software (PLGS, Waters Corporation, Waters S.p.A., Sesto San Giovanni, Italy). The qualitative identification of proteins was obtained by searching the *Mus musculus* database (UniProt KB/Swiss-Prot Protein Knowledgebase restricted to *Mus musculus* taxonomy) to which the sequence from *Saccharomyces cerevisiae* enolase (UniProtKB/Swiss-Prot AC: P00924) was appended.

Search parameters were set as the following: automatic tolerance for precursor ions and for product ions, minimum three fragment ions matched per peptide, minimum seven fragment ions matched per protein, minimum two peptides matched per protein, one missed cleavage, carbamydomethylation of cysteines and oxidation of methionines as fixed and variable modifications, and false positive rate (FPR) of the identification algorithm under 1% and 100 fmol of the enolase set as calibration protein concentration. The most reproducible proteotypic peptides for retention time and intensity of enolase digestion (*m*/*z* 745.43, *m*/*z* 814.49, *m*/*z* 1288.70, *m*/*z* 1416.72, *m*/*z* 1578.80, and *m*/*z* 1840.89) were used to normalize the table of EMRTs. The expression analysis was performed considering technical replicates available for each experimental condition (i.e., untreated and treated with H_2_S, three biological replicates, four technical replicates) following the hypothesis that each group was an independent variable. The identification of protein was based on the detection of more than two fragment ions per peptide, and more than two peptides measured per protein. The list of normalized proteins was screened according to the following criteria: protein identified in at least three out of four runs of the same sample with a fold change of regulation higher than ±20%; only modulated proteins with 0 < *p* < 0.05 were considered significant.

### 2.4. Protein Ontologies and Network Analysis

To identify biologically relevant molecular pathways, the proteomic datasets were analyzed using a bioinformatic analysis tool based on QIAGEN’S Ingenuity Pathway Analysis (QIAGEN’S Ingenuity Pathway Analysis, Ingenuity Systems, http://www.qiagen.com/ingenuity). This allowed exploring functional associations relevant to the experimental results. The analysis parameters were set as the following: direct and indirect relationships, endogenous chemical substances included, all molecules and/or relationships considered as the summary filter. The most significant categories associated with the loaded datasets were identified by calculating their significance (*p*-value, Fischer test). A *p*-value threshold was set at 0.05, which showed the probability of association between genes/proteins present in the datasets and each pathway (canonical pathway, top tox list, and biological function).

### 2.5. Toxicity Experiments

Spinal-cord cultures were exposed to the H_2_S donor, Na_2_S (SIGMA-Aldrich, Milan, Italy), following the same protocol described in Davoli et al., [1]. All experiments were performed between 10 and 12 days in vitro (DIV). For imaging experiments, cells were plated on 13-mm cover glasses. At 10 DIV, the cells were pre-exposed to the BAX Inhibiting Peptide V5 (SIGMA-Aldrich, Milan, Italy; 50 µM) or necrostatin-1 (Merck S.p.a., Roma, Italy; 5 and 15 µM) for 1 h, and then co-exposed to 100 µM and 200 µM Na_2_S for 18 h. After 18 h, the cells were fixed in paraformaldehyde (4%). To visualize motor neurons, cultures were stained with SMI-32 (Covance, Princeton, NJ, USA; 1:1000), and nuclei were stained with Hoechst 33342 (SIGMA-Aldrich, Milan, Italy). SMI-32-positive cells were quantified by direct counting, and their number was normalized to the untreated spinal-cord preparation. For toxicity experiments, we performed a Student’s *t*-test, setting the significance to *p* < 0.05.

## 3. Results

### 3.1. Proteome Profiling Using Label-Free Proteomics Analysis

To understand the effects of H_2_S-mediated toxicity on neuronal cultures, we performed a deep proteomic investigation in order to obtain the expression profile of proteins whose levels change in response to treatment with Na_2_S. Na_2_S, like NaHS, is water-soluble, and is widely used in experimental conditions as an H_2_S donor [18,19,20].

To investigate the different protein profiles with and without Na_2_S, we used protein extracts from spinal-cord cultures not treated and treated with Na_2_S at a concentration of 200 µM, in which the cytotoxicity of motor neurons was markedly enhanced, as we previously demonstrated in Davoli et al. [1]. The comparative proteome analysis was performed using MS^E^ isotope label-free profiling. Quality control measures were performed on the analytical replicates to determine the analytical reproducibility of the mass measurement and chromatographic retention time of each peptide (data not shown).

As thoroughly described in Section 2, using this approach, we identified a total of 316,278 molecular spectral features (EMRTs) and 209 differentially expressed proteins across both conditions of the experimental dataset (not treated vs. Na_2_S).

### 3.2. Expression of Inflammation and Oxidative Stress-Related Pathways Is Modified after H_2_S Treatment

To gain a comprehensive view of the putative cellular and molecular networks in which the identified proteins might be involved, we analyzed our proteomic datasets with a bioinformatic approach.

According to the parameters described in the Section 2, QIAGEN’s Ingenuity Pathway Core Analysis revealed inflammation-related pathways that were most significantly perturbed in the dataset of interest. Particularly, this unbiased systems-biology approach identified significant differential expression of proteins involved in the Nrf-2-mediated oxidative stress response canonical pathway (*p* = 4.30 × 10^−11^). Likewise, the Nrf-2-mediated oxidative stress response was identified as one of the most altered among the top toxic pathways (*p* = 4.98 × 10^−14^). Similarly, pathways related to cell-death processes, among the disease-related pathways, were shown to be perturbed (Table 1). In more detail, Table 2 shows the proteins related to the Nrf-2-pathway and those differentially expressed under both conditions. In particular, a high expression of peroxiredoxins was highlighted, and is discussed below (Table 2).

### 3.3. Hydrogen Sulfide Toxicity Operates through Apoptotic and Necroptotic Pathways

**Figure 1:**
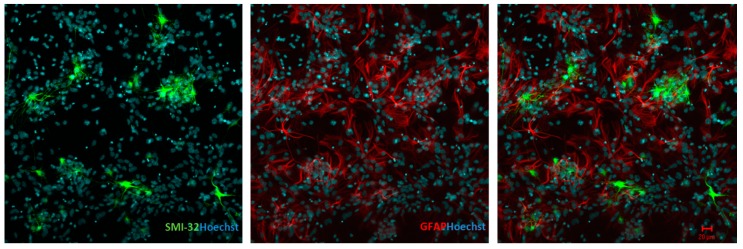
Spinal-cord mixed-culture system at seven days showing immunostaining for SMI-32 (left; green) and GFAP (center; red). The merge is shown in the right panel. Hoescht 322 staining is shown in blue. Scale bars indicate 20 μm.

Figure 1 is a representative image of the mixed-culture system that we utilized in our experiments. Spinal-cord cultures were probed with anti-neurofilament H non-phosphorylated (SMI-32, motor neuron; green) and glial fibrillary acidic protein (GFAP, astrocytes; red). We used this system since the interactions between astrocytes and neurons in co-cultures are an important factor for neuronal well-being. Astrocytes are known to release factors that can activate endogenous protective [21] or harmful [22] mechanisms for the neurons.

**Figure 2:**
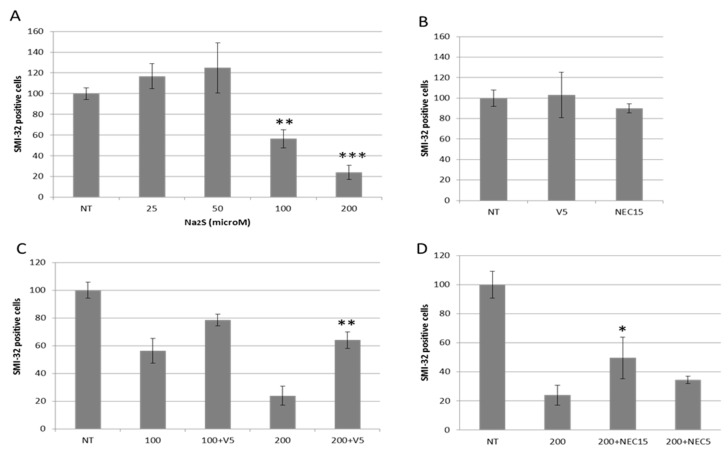
Na_2_S toxicity in spinal-cord cultures. (**A**) Mixed spinal-cord cultures were exposed to 25, 50, 100, and 200 µM Na_2_S. (**B**) V5 50 µM and necrostatin 15 µM were tested alone for their toxicity on spinal-cord cultures. (**C**) The cultures were exposed to 100 and 200 µM Na_2_S plus 50 µM V5, and (**D**) to 200 µM Na_2_S plus necrostatin (NEC) 5 and 15 µM. After 18 h, the number of surviving cells was assessed by direct counting of SMI-32-positive cells, and was normalized to the untreated (NT) values. Data are presented as percentages and as the mean ± standard error of the mean (SEM) from three experiments counted in triplicate. The values were compared using a *t*-test analysis. *** *p* < 0.001 vs. NT (**A**), ** *p* < 0.01 vs. 200 µM Na_2_S (**A**,**C**), * *p* < 0.05 vs. 200 µM Na2S (**D**).

Figure 2A shows the cytotoxic effects of the H_2_S donor, Na_2_S (sodium sulfide), on motor neurons, confirming NaHS (sodium hydrosulfide) toxicity, as we previously reported [1]. Cytotoxicity of motor neurons due to Na_2_S was markedly enhanced at the 100 µM and 200 µM concentrations, with the 200 µM concentration reaching a death rate of almost 80%, while the same concentrations of Na_2_S were not toxic to GFAP+ and Iba-1+ cells (not shown). We then asked whether H_2_S-mediated toxicity operated through the activation of apoptosis, and specifically a Bax-mediated mechanism [23]. Cultures were pretreated with 50 μM V5, a synthetic Bax inhibitor peptide, as described in and used by Nagai et al. [21], and then, cultures were co-exposed to 100 µM and 200 µM Na_2_S for 18 h. Figure 2C shows the ability of the Bax inhibitor to revert, at least in part, the death of the SMI-32+ neurons. Its efficacy was clearer against the 200 µM Na_2_S concentration (from 24 ± 7 to 64 ± 6), although V5 was also capable of increasing SMI-32+ survival rate by nearly 40% (from 56 ± 9 to 79 ± 4) vs. the 100 µM concentration.

Necroptosis is a newly described form of regulated cell death. It is a regulated form of necrosis which is dependent on the serine/threonine kinase receptor-interacting protein 1 (RIP1) [24]. It is specifically inhibited by a small molecule, necrostatin-1 (Nec-1), which targets RIP1. It was proposed as an additional noxious pathway in ALS-related motor neuron death [25,26]. Hence, to explore its involvement in the H_2_S-mediated SMI-32+ neurons, we treated the primary culture under Na_2_S toxicity (200 µM) with Nec-1 at two different concentrations, 5 µM and 15 µM. Nec-1 protection was not as effective as the V5 treatment; however, it was still able to compete against Na_2_S-mediated toxicity, from 24 ± 7 to 50 ± 14 for the 15 µM concentration, and from 24 ± 7 to 34 ± 2 for the 5 µM concentration (Figure 2D). V5 and Nec at 15 μM only were not toxic to the SMI-32+ neurons (Figure 2B). These results indicate that both pathways, apoptosis and necroptosis, contribute to H_2_S-induced motor neuron death.

The probing of the cultures with fractin, a specific apoptotic marker [27] of actin fragmentation as a result of caspase-3 activation [28], showed a general increase of its immunopositivity in the cultures (not shown).

## 4. Discussion

In the presented study, we analyzed the effects of H_2_S toxicity on mixed primary spinal-cord cultures. Moreover, in order to investigate the changes in protein pathways caused by H_2_S treatment, we used proteomic analysis based on the MSE isotope label-free approach. The data presented here provide evidence that H_2_S-mediated toxicity triggers the activation of Bax-dependent and necroptosis-dependent pathways. The proteomic analysis indicated that primary spinal-cord cultures respond to H_2_S-mediated toxicity by altering pathways related to the oxidative stress response.

We showed that V5, a cell-permeable apoptosis inhibitor pentapeptide (Val-Pro-Met-Leu-Lys), is effective in protecting motor neurons from Na_2_S-mediated toxicity. A member of the Bcl-2 family of proteins, Bax, is a silent protein that resides in the cytosol. In response to apoptotic stimuli, Bax translocates to the mitochondrial outer membrane, causing the release of apoptogenic factors, like cytochrome c, which, together with the apoptotic protease-activating factor-1 (Apaf-1), forms a critical component of the apoptosome [29,30,31,32,33]. This complex leads to the activation, in an ATP-dependent manner, of caspase-9, which then cleaves and activates caspase-3 and -7 [34].

Recently, an additional programmed cell-death pathway emerged, named necroptosis, which is a form of cell death characterized by an increase in the volume of the cell, organelle swelling, and rupture of the plasma membrane [35]. Necroptosis is considered as an alternative to apoptosis, since it is typically induced when caspase activity is blocked, yet recent evidence indicates that Bax and Bak, responsible for the induction of apoptotic cell death, are involved in the induction/regulation of necroptosis [36,37,38], although these data are still debated [39,40]. In order to evaluate the contribution of this pathway to the mechanism of action of H_2_S, primary spinal-cord cultures were incubated with Na_2_S in the presence of two concentrations of Nec-1. The targeting of RIP1 was able to partially rescue the SMI-32+ cells, particularly at the highest concentration used (15 μM). This result indicates that necroptosis contributes, together with apoptosis, to H_2_S-induced cell insult, and is involved in its cellular toxicity.

The proteomic profile of spinal-cord cultures treated with Na_2_S here shows that its treatment provokes an upsurge of both oxidative stress and inflammatory pathways (Table 1). We identified, of particular interest, the Nrf-2-mediated oxidative stress response, peroxiredoxins, and hypoxia-inducible factor signaling.

Nrf-2 is one of the main regulators of the antioxidant response. In a state of oxidative stress, it binds to and upregulates the antioxidant response elements (ARE) [41,42], and, in doing so, it triggers the cellular antioxidant and anti-inflammatory defense, and strengthens the mitochondrial protection [43]. The ARE-driven genes are a group of antioxidant and detoxifying enzymes that, when activated, oppose cellular damage in many different tissues and organs [44]. Nrf-2 is linked to several neurodegenerative diseases. In animal models of Huntington’s and Parkinson’s diseases, the ablation of Nrf-2 worsened cell death [45,46]. Johnson and co-workers, using a transgenic mouse with Nrf-2 expression restricted to astrocytes, reported a significant delay in disease onset and extended life span in a familial model of ALS [47]. Interestingly, the same group reported that, when Nrf-2 is selectively expressed in neurons or type II muscle fibers, only a delayed onset, but not a longer survival, was observed [48]. This further highlights the fundamental role that astrocytes have in the onset and, eventually, in the treatment of ALS as a pharmacologically targeted cell type. Free-radical damage or abnormal free-radical metabolism is widely described in ALS in post-mortem human Central Nervous System tissues and in CSF from ALS patients [49,50]. Moreover, oxidative damage to proteins, lipids, and DNA was described in tissues from sporadic ALS patients and Sod1-linked familial ALS [51,52]. We may infer that the increase in Nrf-2 protein and related pathways under Na_2_S treatment is an attempt to cope with the toxic environment created by the increase in oxidation and reactive oxygen species (ROS). Interestingly, in our previous work [1], we reported that H_2_S is mainly a glial product. Nrf-2 and ARE-driven genes co-localize with reactive astrocytes in the symptomatic SOD1G93A mouse spinal cord [53]. In the context of a chronic pathology, the cell may activate multiple, even redundant, systems to cope with inevitable death, such as the over-production of H_2_S as an anti-inflammatory and anti-apoptotic bio-product, and Nrf-2 as a regulator of the antioxidant response. Hence, while H_2_S treatment affects the number of SMI-32+ neurons, causing their death, it also triggers the Nrf-2-mediated pathway, which might be seen as an attempt by the surviving cells to cope with H_2_S toxicity, by setting off an antioxidant response.

Our proteomic analysis also underscored a significant increase in the expression of the peroxiredoxins (PrXs), PrX1, PrX2, and PrX6, in H_2_S-treated cultures compared to the untreated cultures (Table 2). High concentrations of H_2_S are pro-inflammatory. We already demonstrated that a pro-inflammatory lipopolysaccharide (LPS) challenge stimulates the production of H_2_S in primary spinal-cord cultures [1]. It was speculated that the increased expression of several PrXs in inflammatory situations could be a negative feedback loop response to protect cells against oxidative insult, but could also contribute to the control of redox-sensitive effectors [53,54]. For example, an immunohistochemical study performed on post-mortem sporadic and superoxide dismutase 1 familial ALS human specimens and SOD1 familial ALS mouse models reported that the number of neurons negative for PrX2/glutathione peroxidase-1 (GPxl) increased with ALS disease progression [55]. The authors concluded that the breakdown of this redox system at the advanced disease stage accelerates neuronal degeneration and/or the process of neuronal death. PrXs are regulators of the redox system that is one of the most crucial supporting systems in neurons. PrXs together with glutathione peroxidase-l (GPxl) form an antioxidant defense system synchronously linked to other important cell-supporting systems. PrXs are major players in inflammatory processes since they act as cytoprotective enzymes against elevated concentrations of ROS/reactive nitrogen species (RNS) generated during inflammation, as well as modulators of redox signaling to control, for example, the synthesis and release of inflammatory mediators. They have a clear protective role in inflammation. The increased expression of PrXs could indicate the ability of the surviving cells (for example, the glia population) to activate these protective factors. 

Hypoxia-inducible factors (HIFs) are transcription factors that recruit a number of co-activators inside the nucleus, controlling the expression of a series of target genes associated with the control of oxygen homeostasis [56]. HIFs are master regulators of cellular responses. In particular, hypoxia-inducible factor-1 (HIF-1), a heterodimer made of the inducible HIF-1α subunit and the structural HIF-1β subunit [57,58], is a transcription factor linked to the activation of more than 100 genes involved in angiogenesis, glucose metabolism, cell survival, and metastasis [59,60]. While under normoxia, the HIF-1α gene is continuously transcribed and translated, while in hypoxic conditions, the HIF-1α protein accumulates [61]. H_2_S can substitute oxygen as a substrate of the mitochondrial respiratory chain; however, when its concentration reaches high levels, it becomes a poison and blocks complex IV, leading to a decrease in ATP production, mitochondrial malfunction, and inevitable cell death [62]. Its role in the regulation of HIF-1 factors is somehow elusive, and it varies depending on cell type and experimental conditions [63]. In rat brain capillary endothelial cells, it was described that NaHS increases expression of the HIF-1α protein and binding activity under CoCl_2_-induced hypoxia-mimetic conditions [64]. In contrast, NaHS inhibits HIF-1 activation in human hepatoma Hep3B cells, human cervical carcinoma HeLa cells, and human aortic smooth-muscle cells [65]. Our proteomic data indicate that, in our cellular system, H_2_S treatment increases HIF signaling. 

## 5. Conclusions

Taken together, the evidence presented here indicates that the over-production of H_2_S activates pathways linked to oxidative stress and inflammation. Moreover, we showed that inhibition of the Bax-related pathway can effectively decrease H_2_S-mediated motor neuron death.

Further analyses are needed to understand the role of H_2_S in spinal-cord homeostasis, and its interaction with inflammation and oxidative stress-related pathways.

## Figures and Tables

**Table 1:** QIAGEN’S Ingenuity Pathway Analysis: top canonical pathway, disease and biofunctions, and molecular and cellular function.

**Canonical Pathway**	***p*-Value**
PI3K/AKT Signaling	1.54 × 10^−12^
Cell Cycle: G2/M DNA Damage Checkpoint Regulation	3.60 ×10^−11^
Nrf-2-mediated Oxidative Stress Response	4.30 × 10^−11^
**Top Tox Lists**	***p*-Value**
Nrf-2-mediated Oxidative Stress Response	4.98 × 10^−14^
Oxidative Stress	4.63 × 10^−11^
Hypoxia-Inducible Factor Signaling	3.60 × 10^−3^
**Top Diseases and Biofunctions**	***p*-Value**
Free-Radical Scavenging	2.89 × 10^−4^–2.50 × 10^−8^
Small-Molecule Biochemistry	3.21 × 10^−4^–2.50 × 10^−8^
Cell Death and Survival	7.12 ×10^−4^–3.22 × 10^−8^

**Table 2:** Differentially expressed proteins in untreated (NT) extracts and in those treated with Na_2_S, identified using label-free nUPLC-MS/MS. ^1^: Protein sequence identifier according to the UniProtKB/Swiss-Prot Protein Knowledgebase; ^2^: protein name; ^3^: ProteinLynx Global Server (PLGS) score; ^4^: protein found highly represented in NT or NaHS extracts; ^5–7^: ratio of expression between NT and Na_2_S.

Accession ^1^	Description ^2^	Score PLGS ^3^	Highly Expressed ^4^	NT:NaHS Ratio ^5^	NT:NaHS Log(e) Ratio ^6^	NT:NaHS Log(e) Std Dev ^7^
*Q9CQV8*	14-3-3 protein beta/alpha	2336.96		1.33	0.29	0.11
*P62259*	14-3-3 protein epsilon	992.11		1.03	0.03	0.19
*P61982*	14-3-3 protein gamma	832.06		1.43	0.36	0.12
*O70456*	14-3-3 protein sigma	684.53		1.03	0.03	0.24
*P68254*	14-3-3 protein theta	895.91		1.24	0.22	0.11
*P20029*	78 kDa glucose-regulated protein	635.95		1.12	0.11	0.09
*P60710*	Actin, cytoplasmic 1	339.07		0.94	−0.06	0.02
*P07901*	Heat-shock protein HSP 90-alpha	833.68		1.25	0.23	0.1
*P11499*	Heat-shock protein HSP 90-beta	1344.62		1.15	0.14	0.07
*P35700*	Peroxiredoxin-1	3181.26	NaHS			
*Q61171*	Peroxiredoxin-2	898.73		0.9	−0.1	0.08
*O08709*	Peroxiredoxin-6	566.13	NaHS			0.09
*P08228*	Superoxide dismutase [Cu-Zn]	572.41		0.9	−0.06	0.03
*O54790*	Transcription factor MafG 1	363.28	NaHS			
*P20152*	Vimentin	23,689.85		0.77	−0.26	0.04
